# Quality Assessment of Serially Ultradiluted and Agitated Drug *Digitalis purpurea* by Emission Spectroscopy and Clinical Analysis of Its Effect on the Heart Rate of Indian *Bufo melanostictus*

**DOI:** 10.1155/2013/571464

**Published:** 2012-12-27

**Authors:** Anup Sharma, Bulbul Purkait

**Affiliations:** ^1^B.C.R.T. Hospital and School of Medical Science and Technology, Indian Institute of Technology Kharagpur, C-1/164, Kharagpur, West Bengal 721 302, India; ^2^Department of Biochemistry, Midnapore Medical College and Hospital, West Midnapore West Bengal 721101, India

## Abstract

The investigation of ultradiluted (homeopathic) drugs is extremely interesting and challenging, and from that point of view this study shows novelty. A study of in vivo changes in heart rate of the Indian *Bufo melanostictus* caused by commercially available serially ultra-diluted and agitated extract of *Digitalis purpurea* has been tried in order to understand their pharmacological role. RR interval (of ECG) was compared after intraperitoneal administration of serially diluted and agitated *Digitalis purpurea* extract, diluent rectified spirit, and Digoxin in anesthetized animals. The study revealed statistically significant changes in the heart rate after application of these drugs except in case of Digoxin and the 200th serial dilution of *Digitalis purpurea*. The duration of RR intervals after application of the drugs was corroborative of the effect of Digoxin and *Digitalis purpurea* extract up to 30th dilution. Emission spectra were obtained for the experimental ultra-diluted *Digitalis purpurea* extract and Digoxin to identify and characterize them. The observed RR pattern and emission spectra show an association. The quality assessment of the commercial ultra-diluted organic drugs obtained from natural products may be initiated by monitoring in vivo studies on animal models.

## 1. Introduction

Exploration of in vivo effect of commercially available serially ultradiluted (with rectified spirit) and agitated drugs (SAD) is required [1], to understand their pharmacological role. Although “drug proving” studies of the effect of ultradiluted drugs on humans have been done, these studies did not deliver enough supporting laboratory evidence to show its relationship with ultradiluted drug. Myths regarding ultradiluted drugs continue. Myths are that homeopathic drugs available readily across the counter, specially the ultradiluted ones, are placebo and do not cause physiological and toxicological changes. The medical and scientific fraternity cast aspersions on the role of homeopathic medicine because of paradigmatic differences [1]. Hence, the assessments of the quality of SAD in terms of their physiological and toxicological effectiveness are aspects that need attention. The investigation of homeopathic drugs is extremely interesting and challenging. This motivated us to take up the study, which was conducted in two parts: first, an in vivo observation of the cardiac effect produced by the serially ultradiluted and agitated *Digitalis purpurea *extract and the purest known comparable drug Digoxin and,second, the fluoroscopic analysis of these drugs by identification of medicinally active ingredients in them. Small animal, the Indian *Bufo melanostictus *(IBM), was chosen as the subject in which the changes in the heart rate (HR) were recorded through digital ECG signals produced after application of intraperitoneal injection of the natural cardiotropic SAD *Digitalis purpurea, *Digoxin, and rectified spirit (91.4%). Electrocardiograph signal was acquired by using special electrodes placed on limbs of the IBM. The in vivo study was considered unique for qualitative assessment of the role of level of serial dilution of *Digitalis purpurea* extract in producing changes in the heart rate (RR). The emission spectroscopy of SAD *Digitalis purpurea *and Digoxin added analytical dimension to the work.

RR represents the ventricular interbeat interval, recordable in vivo [2]. No work showing ECG recording of an IBM is available. This study would be important in drug research of complementary and alternative medicine (CAM), experimental drug, veterinary science, toxicology, and cardiology. 

Practitioners of different systems of medicine with paradigmatic differences have used *Digitalis purpurea *and the like in varying concentration dilution, to treat patients for clinical conditions having manifestation contrary to each other. In modern medicine “Digitalization” is used to reduce the heart rate, while practitioners of homeopathy use it in ultradiluted doses in cases having low heart rate. Details of use of ultra-dilution of *Digitalis purpurea* extract in clinical conditions with low heart rate are given in Homeopathic Materia Medica Pura written around 1833, by founder of homeopathy Hahnemann and Lectures on Homoeopathic Materia Medica by James Tyler Kent in 1905, The Study of Materia Medica by C.M. Boger in 1908 and many more of such literature. William Withering started using decoction of *Digitalis purpurea *for “Dropsy” since December 8, 1775 and undertook many such applications thereafter. 

Anything more diluted than microvolume (10^−6^), that is, as a concentration of one microliter or microgram of solute in one liter of solvent, is considered as “ultra-dilution.” 

In SAD, plant extracts of drugs, say *Digitalis purpurea*, indicated by “*θ*” or “*Q*” are diluted serially to 1: 99 with rectified spirit (91.4%) followed by giving ten strokes/jerks (succussion) to the container. This level of diluted attenuation is called 1st potency, denoted by 1 or 1c. In this dilution scale, the first potency should contain 1/100th part of the original drug. Next potency 2c will contain 1/100th part of the 1st potency, and so on. *Digitalis purpurea *“1c” is diluted to the level of 1x*E* − 2; the dilution “30” contains about 1x*E* − 60 of ingredients, and so on. SAD dilutions in other scales have not been used in this work, and for brevity, they are not described here. In biological fluids, it is not yet possible to measure *Digitalis purpurea *in 6, 30, and 200 or other ultradilutions.  Table 1 shows computed level of dilution of SAD drugs.

The objectives of the study were (a) to observe the effect of commercially available SAD (homeopathic drug) *Digitalis purpurea *(in different dilutions) and Digoxin on the heart rate of small animal Indian* Bufo melanostictus*, (b) to compare the effects produced by (homeopathic drug) *Digitalis purpurea *in different dilutions and Digoxin, and (c) to identify and characterize the experimental drugs by fluorescence spectroscopy.

IBM is not an endangered species and serves as food for some tribes in West Bengal, India. A preliminary comparison between HR of IBM, rabbits, and albino rats was made practically to find out the suitability of the animal species for the present research. Data related to albino rats and rabbits are not produced in this paper for brevity. In the study, IBM was found to be susceptible to *Digitalis purpurea*. It has a normal heart rate that may be comparable to humans, does not require sacrifice for this experimentation, and can be anesthetized easily, and the IBM recovers well after the experiment. Isolation of IBM heart and additional temperature control for maintenance of heart are not required for recording its ECG in a noninvasive method. These conditions allow near-natural and comparable parameters with the humans. In order to avoid impact of climate and weather change, a complete set of studies were conducted in one rainy season. During rainy season, IBMs are healthy, active, their metabolic function is at its best, and they have consistent HR.

## 2. Methodology

The Institutional Ethical Committee for Research on Humans and Animals (IEC) of Indian Institute of Technology, Kharagpur, India, provided necessary ethical clearance and infrastructural support to conduct the experiment as per the prevalent guidelines for animal experimentation in laboratory. Dedicated electrical earthing was installed in the animal house and in the entire laboratory. A veterinary surgeon was engaged to give guidance on all the aspects of animal facility, care, and for conducting research. SAD drugs (prepared as per Indian Homeopathic pharmacopoeia) and contamination-free double-distilled Rectified Spirit (91.4%) were procured from an authorized manufacturer, M/s Hahnemann Publishing Company, Kolkata, India. *Digitalis purpurea Q* or *θ* was of batch no. 6725 of September 2005, *Digitalis purpurea *6, *Digitalis purpurea *30, and *Digitalis purpurea *200, all from the same batch of June 2005. Double-distilled water was used in the experiment. Merck make Diethyl ether was used for inducing general anesthesia. Inj *Digoxin*, 25 mg/mL, manufactured by Samarth Pharma was used. Electrocardiogram and tailor-made software of Recorders and Medicare System, India, was used to record and measure digital cardiac signals. Good conducting electrodes, suitable for the limbs of IBM, were specially designed and manufactured for this experiment. Animals, procured from authorized vendors, were screened for general health conditions and injury marks. Cardiac condition of the IBM was assessed using highly sensitive cardiophonic stethoscope. Simultaneously, neural reflexes were monitored. Stabilized healthy IBMs, of medium size, weighing ~ 120–200 grams, were randomly selected for each set of experiments. Stabilized IBMs were acclimatized for 2 weeks. IBMs were kept exclusively in very spacious natural surroundings, with natural diet. They were handled gently to familiarize with human interaction, equipment, and accessories in the animal house and laboratory. Entry of outsiders was prohibited, to restrict disturbance to the animals. High standard of personal hygiene and cleanliness was strictly maintained throughout for conducting the research. Prior to the experiment, the IBMs were given a wash with double-distilled water. Each IBM was given general ether inhalation anesthesia till complete flaccidity of the limbs was reached, and stimulus failed to evoke any reflex. Subsequently, on different sets of ether anesthetized IBM 0.5 mL of intraperitoneal injection of either of the drugs, (only one test drug for one animal), Digoxin, *Digitalis purpurea Q* or *θ*, *Digitalis purpurea *6, *Digitalis purpurea *30, and *Digitalis purpurea* 200, and also the diluent Rectified spirit (91.4%), were administered. The same experiment was repeated by inhalation of ether for half a minute more after the complete flaccidity of limbs, to monitor the effect of prolonged use of ether. Highly conducting electrode jelly was applied on the limbs (at the site of attachment of electrodes) of the anesthetized IBM. Electrodes, as per the markings, for example, LA for left front limb, RF for right hind leg, and so on, were put on all the limbs for real-time continuous monitoring and recording digital cardiac signals. ECG was obtained for each case as mentioned previously. Normal ECG signals and ECG showing variations were especially frozen and saved. Each screen could hold and display 10 seconds of recorded ECG signals. The bass filter, frequency for acquiring signal, magnifications, noise reduction, base line correction, calibration, and optimization were set as per recommendation of the software for obtaining digital cardiac signals.

Ten IBMs died during the experiment. Out of this ten, four animals died during deep anesthesia, four animals died after Digoxin was given, one died after getting a dose of *Digitalis purpurea θ*, and one died after getting a dose of *Digitalis purpurea *30. After the experiments the animals were kept under observation for the next three days. When normal activities were noticed, the animals were released safely in the jungles.

From the obtained ECG, RR (in milliseconds) was manually and electronically calculated. To avoid bias, the entire statistical analysis was carried out by a biostatistician and also by an epidemiologist, who were not involved with the laboratory work. Excel, origin, and SPSS were used for the analysis.

**Figure 1:**
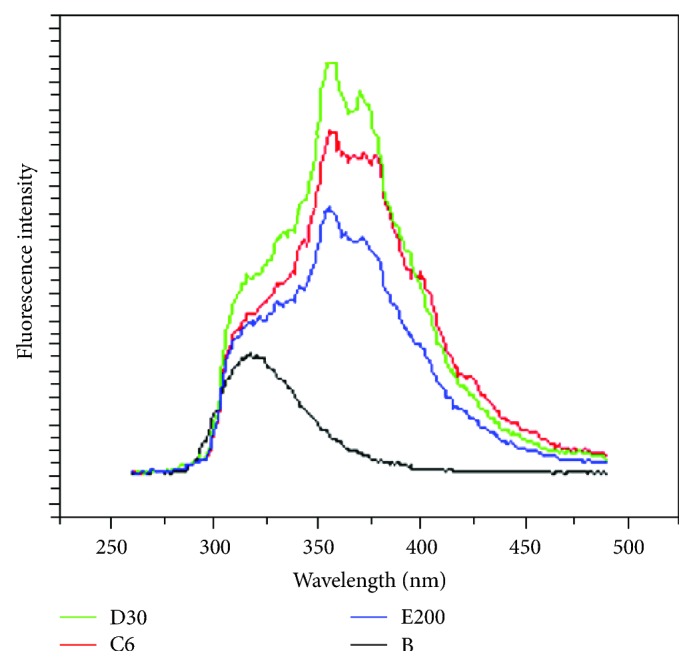
Emission spectra obtained by analytical fluorescence spectroscopy of natural product *Digitalis *purpurea derivative having purest derivative component *Digoxin *(B) showing emission maxima at 318 nm. C6 = *Digitalis purpurea *6 having emission maxima 357 nm and 374 nm. D30 = *Digitalis purpurea* 30 emission maxima 357 nm and 374 nm, E200 = *Digitalis purpurea *200 emission maxima 357 nm and 374 nm. In all the emission studies, the samples are excited at 255 nm.

The fluorescence spectra of the drugs were measured using a Shimadzu (model no UV-1601) spectrophotometer and a Spex-Fluorolog-3 Spectrofluorimeter (model no. FL3-11). The fluorescence spectroscopy was done to detect *Digoxin or Digitalis-like *ingredient in the drugs used and to note its relevance, if any, with the obtained RR. We have taken the fluorescence spectra of the compound at different dilutions. The obtained emission spectra are shown in Figure 1. 

## 3. Discussion

RR data obtained were measured and verified, and appropriations were carried out electronically. The entire readings were crosschecked thrice. Because of software / hardware problem, a few on-screen signals could not be retrieved, and those entries were kept blank. The data which could not be retrieved during verification, as well as the outliers (>±3 standard deviations), were excluded from the data analysis, as they were thought to be biologically implausible values which have shown idiosyncrasies or because of other unknown factors. RR interval data found faulty due to technical and software snag were not considered for statistical analyses. Unequal sample size has appeared in various test groups as it appears in Table 2 because of reasons mentioned previously, malfunctions, and death of animals.

It was also a target to use minimum number of animals for this pilot study. The ANOVA, *P*, and *F* values obtained are given in Table 3.

## 4. Descriptive Data

Since mean is the best estimate of the true value of an experimental quantity, the mean RRs of the IBMs injected with experimental drugs varied as shown in Table 2. The highest mean RR was found to be in the case of Deep Ether Anesthesia (766.22), followed by *Digitalis purpurea *30 (722.18) and Ether Anesthesia (701.97). The lowest mean was found to be for *Digitalis purpurea *200 (582.46) whose standard error (9.83), standard deviation (51.07), and sample variance (2608.39) have been the lowest.

## 5. ANOVA

The one-way ANOVA, given in Table 3, was used in the study to test the hypothesis about the mean (average) of the dependent variable, the RR. Since eight types of drugs were used, the one-way ANOVA was used to find out the differences between (inter) and within (intra) groups. The null hypothesis for ANOVA is that the mean (average value of the dependent variable) is the same for all groups. The alternative or research hypothesis is that the average is not the same for all groups. However, there are exceptions to the rule as is stated in the next section under hypothesis testing.

## 6. Hypothesis Testing

Ho. The null hypothesis: application of different drugs has the same effect in RR changes.
H1. The alternate or research hypothesis: application of different drugs does not have the same effect in RR changes:
Ho: *u*_1_ = *u*_2_ = *u*_3_ =…*u*_*n*_ (Null hypothesis: means of *n* groups are equal),
H1: *u*_1_<or>*u*_*n*_ (Alternative or research: means of *n* groups are not equal).

Since the *F* value 8.02 is greater than 2.07, the results are significant at the 5% significance level. The null hypothesis is rejected, as there is strong evidence that the expected values in the eight groups differ though the *P* value for this test is higher than 0.05. It is concluded that the average of the dependent variable is not the same for all groups.

## 7. Results with Generalized Estimation Equation (GEE)

The effect of different medicinal preparations on the RR intervals (in milliseconds) was seen in the Indian *Bufo melanostictus*. Outliers and omitted readings were not considered in this analysis. The medicines used were analyzed groupwise as given as follows: ether (general inhalation) anesthesia (group = Imedicine_0), ether with double-distilled contamination-free rectified spirit (group = Imedicine_1), ether (producing deep anesthesia) (group = Imedicine_2), ether with *Digitalis purpurea θ* (group = Imedicine_3), ether with *Digitalis purpurea *6 (group = Imedicine_4), ether with *Digitalis purpurea *30 (group = Imedicine_5), ether with *Digitalis purpurea *200 (group = Imedicine_6), and ether with *Digoxin *(group = Imedicine_7).

Linear regression analysis was carried out to see the effect of the drugs on RR interval after adjusting for IBM as a covariate in the equation. The RR interval was treated as a continuous variable with normal frequency distribution. Considering that repeated measurements were made on the same animal after giving the drug, the second analysis was performed considering the data as panel data. The results of linear regression analysis and generalized estimation equation (GEE) are given in Tables 4 and 5, respectively.

Further, considering that repeated measurements were made on a set of animals after giving the drug, the data was treated as panel data set and a GEE was settled. The GEE results are given in Table 5. In both equations, the experimental animal was included as a covariate to nullify influence of interanimal differences.

For the GEE, animals treated with ether anesthesia only were considered as the reference groups. Medicines 1 and 2 are ether with rectified spirit and ether deep anesthesia, respectively. Deep anesthesia had the maximum influence on the frequency of RR (coefficient 132.68; *P* < 0.001). Further with increase in homeopathic dilution of dosage of *Digitalis purpurea *from *θ* to 200 (*θ*, 6, 30, 200), there was a linear increase in coefficients from 22.8 to 49.6 up to potency of 30, but with potency of 200, it dropped to 16.49; however it was not significant (*P* = 0.079). With *Digoxin*, the coefficient was 6.46, and it was not statistically significant. In both groups, the variance was very wide, which may be due to technical difficulty in recording the observations and/or smaller number of observations particularly in case of IBMs injected with Digoxin. However, to establish the effect of *Digoxin *and that of *Digitalis purpurea *200, further experiments have to be conducted in the future. It has also been observed that the type of medication explains only 16% of variance in frequency of RR, thus suggesting that other influencing (confounding) variables should also be taken into consideration to improve explanation of variance in RR. 

From the previous equation, it may be concluded that with increasing potency up to 30 of *Digitalis purpurea*, the coefficient of variance of RR is increasing; however, with *Digitalis purpurea *200, the coefficient of variance of RR is like coefficient of variance of Digoxin, thereby suggesting that RR is significantly influenced by the *Digitalis purpurea*. The observations are in conformity with the equation using linear regression method. 

## 8. Emission Spectra

The emission spectra of *Digitalis purpurea θ*, 6, 12, 30, and 200 and Digoxin were obtained to detect any plausible correlation with the RR results. The primary aim of this work is to detect the presence of *Digoxin* or *Digitalis-like *medicinally active ingredient in the serial ultradilutions of *Digitalis purpurea *using fluorescence spectroscopy, for comparison of the components of the drug which is having intrinsic fluorescence. The fluorescence is likely to be due to *α*, *β*-unsaturated lactone present in the compound. The fluorescence spectrum of the compound is taken at different dilutions in aqueous ethanol [3]. In all the emission studies, the samples were excited at 255 nm [4]. The emission spectra of different dilutions are shown as C, D, and E, and purest medicinally active ingredient Digoxin is shown as B in Figure 1. These fluorescence spectra revealed that Digoxin component shows a structureless fluorescence with emission maxima at 318 nm. The emission spectra of *Digitalis purpurea *6 are shown in Figure 1 as C6.In this case the structureless emission is found to be modified with a partially structured emission with maxima at 357 nm and 374 nm, and further a shoulder at about 318 nm remained which could be due to the original B (Digoxin).The appearance of additional bands at 357 and 374 nm may be ascribed to reduction of self-quenching or dissociation of component substance due to serial dilution. These bands showed an increase in intensity with subsequent dilutions up to a certain level for 30 [5].The serial sequential dilution of the natural product at 6c, 30c, and 200c shows emission maxima at 318 nm rather than the structureless emission as observed with purest Digoxin. This could arise possibly due to the interference caused by other inclusions in the samples. Although, with the dilution, the peak intensity of the compounds should gradually increase or decrease, the gradual increase in emission with dilution is due to reduction of self-quenching of fluorophores as a result of concomitant dilution [6].

The decrease of emission with further dilution is caused probably by simple dilution effect. This is in accordance with the variance in RR observed by use of different sets of drug on IBM. Since the medicinally active ingredients in serial dilution of drug are not detectable by the common analytical methods in vogue, the results cannot be compared with usual standard methodologies. Thus, fluorescence data seem to have some correlation with the results from other techniques [4].

## 9. Conclusion

It is revealed from the emission spectra that Digoxin or Digoxin-like product is present in the commercially available serially ultradiluted* Digitalis purpurea* up to 200. With increasing potency up to 30 of *Digitalis purpurea *extract, the coefficient of variance of RR increases as also the intensity of emission spectra. Intensity of absorption in emission spectra decreases on further serial dilution of *Digitalis purpurea* extract to 200. The result shows a similarity in pattern of variance of RR produced by the experimental drugs and emission spectra obtained from these experimental drugs.

Quality assessment of the commercially available ultradiluted organic drugs obtained from natural products may be initiated by monitoring physiological and toxicological studies on animal models. An advance level of analytical procedure may help in identification of medicinally active ingredient in organic drugs derived from natural plant products.

## 10. Limitations of the Study

Our study has several limitations. It is prospective in nature, and, therefore, it has all the problems associated with such analysis. There are some other components, which are naturally present with *Digitalis purpurea *and may be responsible for its different activities. We cannot exclude the presence of other important predictors of heart rate and variability analysis. Only one statistical method of interpretation that represents this work in true spirit is included. Other than RR, ECG-associated parameters though studied are not included owing to the confines of the paper. Medicinally active ingredients of SAD are difficult to detect analytically in laboratory. SAD is not detectable in biological specimen, bringing limits to the design of the research. The drug *Digitalis purpurea θ* was not diluted for improved physicochemical analytical studies; such dilutions are not found in commercial circulation. The aspects dealing with dilution factor have not been included. The statistical analyses with respect to the toxicological finding/death of animals have not been given.

## 11. Future Scope

It remains to be seen if any change appears in configuration, dilution, and effectiveness due to ultra-dilution and agitation of drug, which may be directly linked to fluorescence data. 

Further analysis of fluorophores from the drug *Digitalis purpurea *and Digoxin may provide in-depth understanding of the components that can bring changes in heart rate. In turn, fine tuning of the heart rate may become possible without any side effect of the drugs like *Digitalis *or similar ones. The diluted drug *Digitalis purpurea θ* may be tried for improved physicochemical and analytical studies. This opens up a vast research area for exploration of in vivo study of SAD/ultradiluted drugs.

## Figures and Tables

**Table 1:** Computed dilution level for potencies 1c, 2c, 3c, and 6c.

Level of dilution	Portion of diluent (by volume)	Dilution level of drug
1c	99	1
2c	1 part of 1c + 99 part diluent	0.01
3c	1 part of 2c + 99 part diluent	0.0001
6c	1 part of 5c + 99 part diluent	1.00*E* − 10

**Table 2:** Summary of statistical data of drugs, numbers of IBMs used, RR, mean, SD, standard error, and lower and upper limits. DP = *Digitalis purpurea*, *θ*, = extract of SAD concentrations are 6, 30, and 200, E_RS = rectified spirit (91.4%), E_DI = Inj. Digoxin, E_DA = ether-induced deep anesthesia, and E_A = ether-induced general anesthesia.

Drug	Count	Mean	Std. error	Median	Std. Dev.	Sample variance	Min	Max	Lower limit (con. level 95%)	Upper limit (Con. level 95%)
DP_*θ*	19	629.87	30.90	581.69	134.69	18141.86	439.26	921.43	569.31	690.44
DP_6	33	614.61	15.37	606.98	88.27	7791.94	497.66	872.31	584.49	644.73
DP_30	19	722.18	22.67	717.24	98.83	9767.96	593.66	925.69	677.74	766.62
DP_200	27	582.46	9.83	573.16	51.07	2608.39	461.56	679.35	563.19	601.72
E_DI	12	611.89	22.47	624.29	77.83	6057.43	480.83	734.00	567.85	655.92
E_RS	25	692.24	15.61	684.75	78.07	6095.63	534.10	834.71	661.64	722.85
E_DA	6	766.22	49.94	808.89	122.32	14961.75	565.00	878.55	668.35	864.10
E_A	22	701.97	20.65	687.41	96.84	9377.60	576.27	882.67	661.51	742.44

**Table 3:** ANOVA and *P* and *F* values.

Source of variation	SS	df	MS	*F*	*P* value	*F* crit
Between groups	472546.6	7	67506.66	8.022936	2.63*E* − 08	2.06912
Within groups	1304202	155	8414.209			

Total	1776749	162				

**Table 4:** Linear regression analysis. The medicines used were analyzed groupwise as given before. Total no. of obs. = 7382; df = model: 8, Residual: 7373, Prob. > *F* = 0.0000; *R*-squared = 0.1613; Adj. *R*-squared = 0.1604; Root MSE = 148.47.

RR interval	Coef.	Std. Err.	*t*	*P*>|*t*|	(95% conf. interval)	
					Lower limit	Upper limit
Rectified spirit (medicine_1)	56.12259	6.464837	8.68	0.000	43.44966	68.79551
Ether (medicine_2)	138.1445	11.78828	11.72	0.000	115.0361	161.2529
*Digitalis purpurea θ* (medicine_3)	36.68673	7.024809	5.22	0.000	22.91609	50.45736
*Digitalis purpurea *6 (medicine_4)	64.35277	8.299983	7.75	0.000	48.08243	80.62311
*Digitalis purpurea *30 (medicine_5)	70.56156	5.815624	12.13	0.000	59.16127	81.96184
*Digitalis purpurea *200 (medicine_6)	47.93883	9.036935	5.30	0.000	30.22386	65.65381
Digoxin (medicine_7)	8.956708	10.4319	0.86	0.391	−11.4928	29.40622
Bufo no.	−1.359518	0.0621821	−21.86	0.000	−1.481413	−1.237623
cons	731.6598	5.034684	145.32	0.000	721.7904	741.5292

**Table 5:** Generalized estimation equation (GEE). GEE population-average model, number of observations 7382, number of groups 115, Obs. per group: minimum1, Gaussian average 64.2, exchangeable maximum 164, Wald chi2 (8) 1229.85, scale parameter 22113.63, Prob > chi2 0.0000.

RR interval	Coefficient	Std. Err.	*z*	*P*>|*z*|	(95% conf. interval)	
					Min	Max
_Imedicine_1	36.84835	6.552434	5.62	0.000	24.00582	49.69089
_Imedicine_2	132.6837	11.55072	11.49	0.000	110.0448	155.3227
_Imedicine_3	22.86807	7.037224	3.25	0.001	9.075362	36.66077
_Imedicine_4	34.53295	8.590522	4.02	0.000	17.69584	51.37006
_Imedicine_5	49.64217	6.172171	8.04	0.000	37.54494	61.7394
_Imedicine_6	16.4916	9.394104	1.76	0.079	−1.92051	34.9037
_Imedicine_7	6.463783	10.35481	0.62	0.532	−13.83127	26.75884
Bufo no.	−1.169226	0.0631614	−18.51	0.000	−1.29302	−1.045432
_cons	740.6548	6.165059	120.14	0.000	728.5715	752.7381

## References

[1] Rao M. L., Roy R., Bell I. R., Hoover R. (2007). The defining role of structure (including epitaxy) in the plausibility of homeopathy. *Homeopathy*.

[2] Di Rienzo M. (1999). *Methodology and Clinical Applications of Blood Pressure and Heart Rate Analysis*.

[3] Mohan J. (2004). Ultra violet spectroscopy. *Organic Spectroscopy Principles and Application*.

[4] Sharma A., Thakur A. K., Purkait B. (2010). Identification of medicinally active ingredients in ultradiluted *Digitalis purpurea*: FTIR and Raman spectroscopic studies. *Medicinal Chemistry Research*.

[5] Sharma A., Purkait B. (2012). Identification of medicinally active ingredient in ultradiluted *Digitalis purpurea*: Fluorescence Spectroscopic and Cyclic-Voltammetric study. *Journal of Analytical Methods in Chemistry*.

[6] Lakowicz J. R. (2006). Quenching of fluorescence. *Principles of Fluorescence Spectroscopy*.

